# An Adaptive External Torque Estimation Algorithm for Collision Detection in Robotic Arms

**DOI:** 10.3390/s25206315

**Published:** 2025-10-13

**Authors:** Cheng Yan, Ming Lyu, Yaowei Chen, Jie Zhang

**Affiliations:** College of Automation, Nanjing University of Science & Technology, Xiaolingwei Street, Nanjing 210094, China; ycyc001007@njust.edu.cn (C.Y.); lumtz@163.com (M.L.); 221110011239@njust.edu.cn (Y.C.)

**Keywords:** robotic arm, bayesian estimation, collision detection, torque estimation

## Abstract

As robotic applications rapidly expand into increasingly complex and dynamic environments, greater emphasis is being placed on the intelligence and safety of human–robot collaboration at the task execution level. In shared human–robot workspaces, even the most precise motion planning cannot fully prevent collisions. To address this critical safety concern, we propose a variational Bayesian Kalman filtering-based external torque estimation algorithm that integrates the robot’s dynamic model while avoiding additional system complexity. We begin by reviewing the robot dynamics framework and the classical external torque estimation method based on generalized momentum. We then derive a Kalman filter-based approach for external torque estimation in robotic manipulators and analyze the adverse effects arising from mismatches in process noise covariance. Finally, we introduce a sliding window-based variational Bayesian Kalman filter, which dynamically estimates the current process noise covariance while simultaneously mitigating the accumulation of recursive errors.

## 1. Introduction

In physical human–robot interaction (pHRI) [1,2], quantifying the severity of collisions on the human body provides critical guidance for higher-level systems in managing post-collision events. Haddadin et al. [3,4] systematically investigated this problem through extensive experiments, quantifying the severity of impacts on different parts of the human body. Their results demonstrated that relative velocity, effective mass, and the elastic modulus of the contact surface all influence collision severity, with the relative velocity at impact identified as the dominant factor. In contrast, the contribution of the robot’s reflected mass exhibits a saturation effect [5,6]. Building on this work, Haddadin later introduced the concept of a “collision event pipeline” [7], a generic stage-based framework that decomposes the collision process into multiple phases to facilitate systematic analysis.

Novel robotic components, such as the adaptive soft robotic actuator (ASRA) [8], have also been explored for collision detection, showing promising response speed and accuracy but limited general applicability. A generalized momentum-based collision detection method was first introduced by Haddadin [3] and experimentally validated in various pHRI scenarios. This approach is simple to implement, requires no external sensors, and has demonstrated broad applicability. At its core, the first-order momentum observer (FOMO) estimates external torques by computing momentum residuals, without relying on joint angular accelerations or the inverse of the inertia matrix, thereby substantially reducing sensitivity to measurement noise [9]. Building on this framework, the second-order momentum observer (SOMO) [10] exhibits stronger robustness to velocity noise, albeit at the expense of slower response and without a complete theoretical proof. An improved second-order observer [11,12] enhances dynamic performance by incorporating a feedforward term, which shortens response time but compromises disturbance rejection relative to the standard SOMO. More recently, Liu [13] proposed a higher-order generalized momentum observer that achieves stronger disturbance rejection by adopting a higher cutoff frequency, though at the cost of increased computational burden inherent to higher-order systems.

Kalman filtering has shown strong potential for suppressing noise in external torque estimation. Wahrburg et al. [14] first applied Kalman filtering to robotic manipulators, formulating a state-space model based on generalized momentum that effectively attenuated noise but exhibited limited performance in handling nonlinearities such as friction. Moreover, due to the inherent properties of Kalman filtering, inaccuracies in covariance matrices led to amplified estimation errors. To address these limitations, Yigit et al. [15] employed the extended Kalman filter (EKF), which improved robustness against nonlinear factors including friction, though estimation accuracy remained suboptimal. Wang et al. [16] further advanced this approach by integrating encoder and accelerometer measurements within an EKF framework to estimate link-side states, achieving substantial improvements in estimation precision and dynamic responsiveness. However, the reliance on accelerometers increased both system cost and complexity. More recently, Dong et al. [15] proposed a sliding window variational adaptive Kalman filtering method grounded in an error probability model. By adaptively updating the external force parameters of the ECM within the process model, this approach significantly reduced estimation error without requiring additional sensors. Neural network basis functions combined with barrier Lyapunov functions have been exploited to enhance signal convergence [12], while fuzzy logic systems have been employed to approximate nonlinearities, leading to fuzzy compensation disturbance observers that mitigate the impact of initial values [17,18].

In recent years, extensive efforts have been devoted to adaptive Kalman filtering algorithms and external torque estimation [19,20,21] in robotic systems. A defining feature of adaptive Kalman filters is their ability to provide real-time estimates of noise covariance. For example, Ref. [22] employed perturbation analysis of the discrete-time algebraic Riccati equation (DARE) to examine the sensitivity of Kalman filters to deviations in noise covariance estimates, thereby assessing estimation accuracy. Building on this line of work, Zhenwei Li et al. [23] developed a variational Bayesian maximum correntropy cubature Kalman filter with adaptive kernel bandwidth, where the bandwidth was updated online using Mahalanobis distance. This approach substantially reduced the influence of non-Gaussian measurement noise on the estimation results. However, the method suffers from high computational cost and sensitivity to parameter tuning, which constrains its applicability in broader scenarios.

In this work, we propose a Kalman filtering-based algorithm for estimating interaction torques in robotic manipulators under conditions of uncertain or time-varying process noise covariance. The method employs variational inference to estimate unknown process noise covariances, decoupling noise parameters from manipulator configurations and payload characteristics. At each measurement cycle, the posterior probability density function of the noise covariance is iteratively updated within a closed-form variational Bayesian framework, yielding an approximate Gaussian posterior of the process noise. This enables optimal external torque estimation through Kalman filtering. The approach is experimentally validated on a Franka manipulator and benchmarked against several state-of-the-art methods for external torque estimation in real robotic systems.

## 2. Materials and Methods

### 2.1. System Dynamics of Robotic Manipulators

When a robotic manipulator interacts with the external environment through force exchange, and assuming rigid joints and rigid links, the system dynamics can be described by the following equation of motion [24]:

(1) $$M\left(q\right)\ddot{q}+C\left(q,\dot{q}\right)\dot{q}+g\left(q\right)=\tau =\tau_{m}+\tau_{e}\;,$$

where the position and velocity of the linkage are denoted by $q,\dot{q}\in R^{n}$, where *n* is the number of joints, respectively, and the two variables are the states of the robot. $M\left(q\right)\in R^{n\times n}$ denotes the symmetric positive definite inertia matrix, $\dot{C(q,q)}\in R^{n\times n}$ denotes the Coriolis matrix satisfying passivity, and $g\left(q\right)\in R^{n}$ is the gravity torque vector. The torque generated by the motor $\tau \in R^{n}$ and the external torque $\tau_{e}\in R^{n}$$\tau_{e}$ are the inputs to the system. In practice, the joint angle *q* can be obtained either by multiplying the motor’s actual angle θ by the joint reduction ratio or directly from the joint’s absolute encoder. The joint angular velocity $\dot{q}$ is computed via differentiation of the joint angle, while the motor torque $\tau_{m}$ is derived by multiplying the measured current with a fixed gain K. The external force $\tau_{e}$ corresponds to the mapping of the generalized contact force $F_{ext}$ in Cartesian space to the joint space of the manipulator.

(2) $$\tau_{e}=J_{c}^{T}\left(q\right)F_{ext},$$

From the reformulation of the manipulator dynamics under the momentum concept, we obtain

(3) $$\dot{p}=\tau_{m}+C^{T}\left(q,\dot{q}\right)\dot{q}-g\left(q\right)+\tau_{e}\;,$$

The standard KF provides the optimal linear estimate of system states under the assumption of an accurate model, even in the presence of noisy measurements. To estimate the interactive torque of a robotic arm using the KF, a model of the robotic arm must be formulated. In this context, $\tau_{a}=\tau_{m}+C^{T}\left(\dot{q},q\right)-g\left(q\right)$ and a noise term $w_{p}∼N\left(0,Q_{p}\right)$. The time derivative of momentum can be interpreted as the resultant of all forces acting on the system at that moment. Consequently, the dynamic equation of the robotic arm can be rewritten as

(4) $$\dot{p}=\bar{\tau }+J^{T}f+\omega_{p}\;,$$

Following the design principle of the classical disturbance observer, the driving torque is modeled as a constant, whereas the derivative of the external torque is treated as noise-driven. Set $\dot{f}=\omega_{f}$, $\omega_{f}\;∼N\left(0,Q_{c,f}\right)$, and the system state variables are defined as $x=\left[\begin{array}{c} p\\ f \end{array}\right]$, which can thus be formulated in the following state-space form:

(5) $$\left[\begin{array}{c} \dot{p}\\ \dot{f} \end{array}\right]=\left[\begin{array}{cc} 0_{n\times n} & -J^{T}\\ 0_{n_{ext}\times n} & 0_{n_{ext}\times n_{ext}} \end{array}\right]\left[\begin{array}{c} p\\ f \end{array}\right]+\left[\begin{array}{c} I_{n}\\ 0_{n_{ext}\times n} \end{array}\right]\bar{\tau }+\omega$$

(6) $$p_{meas}=\left[\begin{array}{cc} I_{n} & 0_{n\times n_{ext}} \end{array}\right]\left[\begin{array}{c} p\\ f \end{array}\right]+\upsilon$$

Here, $p_{meas}$ denotes the measured generalized momentum, $\upsilon ∼N(0,R_{c})$ represents the system measurement noise, and by augmenting the system state variable x, it follows that the process noise ω is assumed to follow the normal distribution:

(7) $$\omega \;∼N\left(0,\left[\begin{array}{cc} Q_{c,p} & 0_{n\times n_{ext}}\\ 0_{n_{ext}\times n} & Q_{c,f} \end{array}\right]\right)$$

Here, $Q_{c,p}$ and $Q_{c,f}$ denote components of the system process noise covariance matrix, while $R_{c}$ represents the system measurement noise covariance matrix.

(8) $$x=\left[\begin{array}{cc} p & J\tau_{e} \end{array}\right]$$

(9) $$A_{C}=\left[\begin{array}{cc} 0_{n\times n} & -J^{T}\\ 0_{n_{ext}\times n} & 0_{n_{ext}\times n_{ext}} \end{array}\right]$$

(10) $$B_{c}=\left[\begin{array}{c} I_{n}\\ 0_{n_{ext}\times n} \end{array}\right]$$

(11) $$u=\bar{\tau }$$

(12) $$y=p_{meas}$$

(13) $$C_{c}=\left[\begin{array}{cc} I_{n} & 0_{n\times n_{ext}} \end{array}\right]$$

(14) $$Q_{c}=\left[\begin{array}{cc} Q_{c,p} & 0_{n\times n_{ext}}\\ 0_{n_{ext}\times n} & Q_{c,f} \end{array}\right]$$

Accordingly, the state-space representation can be rewritten as

(15) $$\dot{x}=A_{c}x+B_{c}u+\omega$$

(16) $$y=C_{c}x+\upsilon$$

where $\omega ∼N(0,Q_{c})$ and $\upsilon ∼N(0,R_{c})$. Upon discretization, the equation can be written as

(17) $$x_{k+1}=Ax_{k}+Bu_{k}+\omega_{k}$$

(18) $$y_{k}=Cx_{k}+\upsilon_{k}$$

Discretization of the above equation yields

(19) $$\left[\begin{array}{cc} A & B\\ 0 & I \end{array}\right]=\exp\left(\left[\begin{array}{cc} A_{c} & B_{c}\\ 0 & 0 \end{array}\right]T_{s}\right)$$

(20) $$\left[\begin{array}{cc} M_{11,k} & M_{12,k}\\ 0 & M_{22,k} \end{array}\right]=\exp\left(H\cdot T_{s}\right)$$

(21) $$Q_{k}=M_{12,k}{\left(M_{11,k}\right)}^{T}$$

Meanwhile, the parameters of the system state equation can be expressed as $C=C_{c}$, $R_{k}=\frac{1}{T_{s}}R_{c}$, $H=\left[\begin{array}{cc} A & Q_{c}\\ 0 & -A^{T} \end{array}\right]$, and $T_{s}$ is the sampling time.

### 2.2. Effects of Noise Modeling Inaccuracies on External Torque Estimation

The optimality of the Kalman filter fundamentally depends on accurate knowledge of process and measurement noise covariances. However, in robotic manipulators, process noise varies with configuration and joint velocity, making fixed covariance assumptions unrealistic. Such mismatches reduce estimation accuracy, leading to false collision detections or erroneous external torque estimates that may compromise system safety. Prior work [15,25] analyzed these effects by comparing filter-calculated (FMSE), true (TMSE), and ideal (IMSE) mean squared errors, providing a quantitative view of estimation error under covariance mismatch.

To analyze the performance of the filter under conditions of process noise covariance mismatch, the process noise covariance matrix $Q_{k,k-1}$ is defined as follows:

(22) $$Q_{k,k-1}^{u}=Q_{k,k-1}+\Delta Q_{k,k-1}$$

Here, $Q_{k,k-1}^{u}$ denotes the process noise covariance matrix employed in the filtering equations, $Q_{k,k-1}$ represents the true process noise covariance matrix of the system, and $\Delta Q_{k,k-1}$ denotes the deviation between them. When the practically applied process noise covariance matrix $Q_{k,k-1}^{u}$ is substituted into the Kalman filter framework, the recursive form of the filter can be expressed as

(23) $${\hat{x}}_{k|k-1}^{f}=A_{k,k-1}{\hat{x}}_{k-1|k-1}^{f}$$

(24) $$P_{k|k-1}^{f}=A_{k,k-1}P_{k-1|k-1}^{f}A_{k,k-1}^{\prime }+Q_{k,k-1}^{u}$$

(25) $$K_{k}^{f}=P_{k|k-1}^{f}C_{k}^{\prime }{\left(C_{k}P_{k|k-1}^{f}C_{k}^{\prime }+R_{k}\right)}^{-1}$$

(26) $${\hat{x}}_{k|k}^{f}={\hat{x}}_{k|k-1}+K_{k}^{f}\left(z_{k}-C_{k}{\hat{x}}_{k|k-1}\right)$$

(27) $$P_{k|k}^{f}=\left(I-K_{k}^{f}C_{k}\right)P_{k|k-1}^{f}{\left(I-K_{k}^{f}C_{k}\right)}^{\prime }+K_{k}^{f}R_{k}{\left(K_{k}^{f}\right)}^{\prime }$$

Here, the superscript *f* denotes the output term computed by the filter. According to the recursive equations, at time step *k*, the covariance matrix ${\hat{x}}_{k|k}^{f}$ must be calculated under the current state estimate $P_{k|k}^{f}$. However, due to the substitution of $Q_{k,k-1}^{u}$, the state estimates across two consecutive time steps are given by

(28) $${\tilde{x}}_{k|k-1}^{f}=x-{\hat{x}}_{k|k-1}^{f}=A_{k,k-1}{\tilde{x}}_{k-1|k-1}^{f}+w_{k,k-1}$$

(29) $${\tilde{x}}_{k|k}^{f}=x-{\hat{x}}_{k|k}^{f}=\left(I-K_{k}^{f}C_{k}\right)A_{k,k-1}{\tilde{x}}_{k-1|k-1}^{f}+\left(I-K_{k}^{f}C_{k}\right)w_{k,k-1}-K_{k}^{f}v_{k}$$

In the presence of covariance mismatch $\Delta Q_{k,k-1}$, the covariance matrix $P_{k|k}$ under the ideal mean squared error (IMSE) fails to capture the true estimation error of the Kalman filter, thereby distorting the update of the Kalman gain K.

### 2.3. Variational Inference-Based Adaptive External Torque Estimation Algorithm

The complexity of robotic systems makes process noise difficult to model, which limits the accuracy of Kalman filter-based external torque estimation. To address time-varying and non-Gaussian noise, we employ variational inference to approximate latent variable distributions from historical system data, enabling online estimation of the noise covariance. This adaptive framework allows dynamic parameter adjustment in response to noise variations, thereby improving the accuracy of external torque estimation for robotic systems.

Building on the feasibility of Kalman filtering for external torque estimation in robotic manipulators, the modeling error of $Q_{k}$ ultimately leads to inaccuracies in torque estimation. The problem addressed in this section therefore reduces to deriving an approximate distribution of $Q_{k}$ in order to improve the accuracy of external torque estimation.

Using variational inference, this subsection provides a detailed derivation of the implementation of variational Kalman filtering within robotic dynamics, with the estimation procedure encapsulated into an algorithmic module. It is assumed that the system noise $Q_{k}$ is governed by an independent dynamic model, decoupled from the system state variable $x_{k}$:

(30) $$p\left(x_{k},Q_{k}|x_{k-1},Q_{k-1}\right)=p\left(x_{k}|x_{k-1}\right)p\left(Q_{k}|Q_{k-1}\right)\;.$$

According to the Chapman–Kolmogorov relation, the prior of the system state $x_{k}$ jointly with the process noise covariance $Q_{k}$ is given by

(31) $$p\left(x_{k},Q_{k}|y_{1:k-1}\right)=\int p\left(x_{k}|x_{k-1},Q_{k}\right)p\left(Q_{k}|Q_{k-1}\right)\times p\left(x_{k-1},Q_{k-1}|y_{1:k-1}\right)dx_{k-1}dQ_{k-1}\;,$$

and with the incorporation of the subsequent measurement, Bayes’ theorem yields the posterior distribution

(32) $$p\left(x_{k},Q_{k}|y_{1:k}\right)\propto p\left(y_{k}|x_{k},Q_{k}\right)p\left(x_{k},Q_{k}|y_{1:k-1}\right)$$

Variational inference approximates an intractable posterior with a tractable distribution, reformulating inference as the optimization of a closed-form variational distribution. Recalling the prediction step of the classical Kalman filter,

(33) $$\begin{array}{c} x_{k|k-1}=A_{k}x_{k-1|k-1}\\ P_{k|k-1}=A_{k}P_{k-1|k-1}A_{k}^{T}+Q_{k} \end{array}$$

In this formulation, $A_{k}$ denotes the state transition matrix. The predicted covariance matrix depends not only on the process noise covariance matrix $Q_{k}$, but also on $A_{k}P_{k-1|k-1}A_{k}^{T}$. To fully characterize the predicted covariance, we introduce a latent variable $\theta =A_{k}x_{k-1|k-1}$, leading to the representation $\Sigma =A_{k}P_{k-1|k-1}A_{k}^{T}$.

The process noise covariance matrix $Q_{k}$ is assumed to have a prior governed by an Inverse-Wishart (IW) distribution. According to mean-field theory, one obtains

(34) $$p\left(x_{k},\theta_{k},Q_{k}|y_{1:k}\right)\approx q\left(x_{k},\theta_{k},Q_{k}\right)\approx q\left(x_{k}\right)q\left(\theta_{k}\right)q\left(Q_{k}\right)$$

The inverse Wishart distribution is adopted due to its broad conjugacy, superior adaptability to complex data structures, and clear advantages in covariance estimation compared to other conjugate priors.The inverse Wishart distribution is better suited for high-dimensional covariance estimation in robotic manipulators, while the inverse Gaussian is more appropriate for localized impulsive noise. For brevity, z is used in place of $\left(x_{k},\theta_{k},Q_{k}\right)$, yielding

(35) $$p\left(z|y_{1:k}\right)\approx q\left(z\right)\approx q\left(z_{1}\right)q\left(z_{2}\right)q\left(z_{3}\right)$$

Formally, the divergence between $q\left(z\right)$ and $p\left(z|y_{1:k}\right)$ is defined as

(36) $$\mathrm{KL}\left(q_{z}\left(z\right)\left|\right|p\left(z|y_{1:k}\right)\right)=-\int q_{z}\left(z\right)\log\frac{p\left(z|y_{1:k}\right)}{q_{z}\left(z\right)}dz$$

Consider the *j*-th latent variable $z_{j}$.

(37) $$\begin{array}{c} \mathrm{ELBO}\left(q\right)=\int_{z_{j}}q_{j}\left(z_{j}\right)\log{\tilde{p}}_{j}\left(z_{j},x\right)dz_{j}-\int_{z_{j}}q_{j}\left(z_{j}\right)\log q_{j}\left(z_{j}\right)dz_{j}+\mathrm{const}\\ =\int_{z_{j}}q_{j}\left(z_{j}\right)\log\frac{{\tilde{p}}_{j}\left(z_{j},x\right)}{q_{j}\left(z_{j}\right)}dz_{j}+\mathrm{const}\\ =-\mathrm{KL}\left(q_{j}\left(z_{j}\right)\left|\right|{\tilde{p}}_{j}\left(z_{j},x\right)\right)+\mathrm{const} \end{array}$$

Evidently, in order to minimize the KL divergence, it follows that

(38) $$q_{j}^{*}\left(z_{j}\right)={\tilde{p}}_{j}\left(z_{j},x\right)\propto \exp\left\{q_{-j}\left[\log p(z,x)\right]\right\}$$

Since $q_{j}^{*}\left(z_{j}\right)$ corresponds to the probability density function of $z_{j}$, it follows that $\int q_{j}^{*}\left(z_{j}\right)dz_{j}=1$, and normalization yields

(39) $$\left(\mathrm{normalize}\right)=\frac{\exp\left\{q_{-j}\left[\log p(z,x)\right]\right\}}{\int_{z_{j}}\exp\left\{q_{-j}\left[\log p(z,x)\right]\right\}dz_{j}}$$

Under the convexity assumption, it follows from the properties of the evidence lower bound that the variational inference procedure is guaranteed to converge.

By the evidence lower bound (ELBO) property, we obtain

(40) $$L\left(q_{z}\left(z\right)\right)=\int q_{z}\left(z\right)\log\frac{p\left(z,y_{k}|y_{1:k-1}\right)}{q_{z}\left(z\right)}dz$$

(41) $$\log q\left(x_{k}\right)=E_{q(\theta_{k},Q_{k})}\left[\log p\left(x_{k},\theta_{k},Q_{k},y_{k}|y_{1:k-1}\right)\right]$$

(42) $$\log q\left(\theta_{k}\right)=E_{q(x_{k},Q_{k})}\left[\log p\left(x_{k},\theta_{k},Q_{k},y_{k}|y_{1:k-1}\right)\right]$$

(43) $$\log q\left(Q_{k}\right)=E_{q(x_{k},\theta_{k})}\left[\log p\left(x_{k},\theta_{k},Q_{k},y_{k}|y_{1:k-1}\right)\right]$$

Expanding $\log p\left(x_{k},\theta_{k},Q_{k},y_{k}|y_{1:k-1}\right)$ yields:

(44) $$\begin{array}{c} \log p\left(x_{k},\theta_{k},Q_{k},y_{k}|y_{1:k-1}\right)=\log p\left(x_{k}|y_{1:k-1},\theta_{k},Q_{k}\right)+\log p\left(\theta_{k}|y_{1:k-1}\right)+\\ \log p\left(Q_{k}|y_{1:k-1}\right)+\log p\left(y_{k}|x_{k}\right)\\ =\log N\left(x_{k}|\theta_{k},Q_{k}\right)+\log N\left(\theta_{k}|x_{k|k-1},\Sigma_{k}\right)+\\ \log IW\left(Q_{k}|u_{k|k-1},U_{k|k-1}\right)+\log N\left(y_{k}|H_{k}x_{k},R_{k}\right) \end{array}$$

It is straightforward to derive the approximate posterior distributions of $q\left(x_{k}\right)$, $q\left(\theta_{k}\right)$, and $q\left(Q_{k}\right)$.

(45) $$q\left(x_{k}\right)=N\left\{x_{k}|E\left(\theta_{k}\right),{\left[E\left({Q_{k}}^{-1}\right)\right]}^{-1}\right\}N\left(y_{k}|H_{k}x_{k},R_{k}\right)=N\left(x_{k}|x_{k|k},P_{k|k}\right)$$

(46) $$q\left(\theta_{k}\right)=N\left\{E\left(x_{k}\right)|\theta_{k},A_{k}\right\}N\left(\theta_{k}|x_{k|k-1},\Sigma_{k}\right)=N\left(\theta_{k}|\theta_{k|k},P_{\theta ,k|k}\right)$$

(47) $$\log q\left(Q_{k}\right)=-\frac{1}{2}\left(u_{k|k-1}+n_{x}+2\right)\log\left|Q_{k}\right|-\frac{1}{2}tr\left[\left(U_{k|k-1}+B_{k}\right){Q_{k}}^{-1}\right]+{C_{3}}^{\prime }$$

where ${C_{3}}^{\prime }$ is an indeterminate constant, and by setting $B_{k}=E\left[\left(x_{k}-\theta_{k}\right){\left(x_{k}-\theta_{k}\right)}^{T}\right]$, we obtain

(48) $$\begin{array}{c} B_{k}=\left[E\left(x_{k}\right)-E\left(\theta_{k}\right)\right]{\left[E\left(x_{k}\right)-E\left(\theta_{k}\right)\right]}^{T}+\mathrm{cov}\left(x_{k}{x_{k}}^{T}\right)+\mathrm{cov}\left(\theta_{k}{\theta_{k}}^{T}\right)\\ =\left[E\left(x_{k}\right)-E\left(\theta_{k}\right)\right]{\left[E\left(x_{k}\right)-E\left(\theta_{k}\right)\right]}^{T}+P_{k|k}+P_{\theta ,k|k} \end{array}$$

Under the stated assumption, $q\left(Q_{k}\right)\propto IW\left(Q_{k}|u_{k|k},U_{k|k}\right)$, $u_{k|k}=u_{k|k-1}+1$, $U_{k|k}=U_{k|k-1}+B_{k}$:

(49) $$E\left({Q_{k}}^{-1}\right)=u_{k|k}{U_{k|k}}^{-1}$$

and accordingly, we have

(50) $$A_{k}=\frac{U_{k|k}}{u_{k|k}}$$

The variational Kalman-based external torque estimation method employs a closed-loop iterative scheme to estimate the process noise covariance, which is then incorporated into the momentum-based Kalman filter torque observer. Simulation results demonstrate that employing a Lyapunov-based function improves torque estimation stability and smoothness, thereby enhancing overall system performance. This formulation preserves the linear optimality of the Kalman filter even under covariance mismatch.

## 3. Results

**Figure 1:**
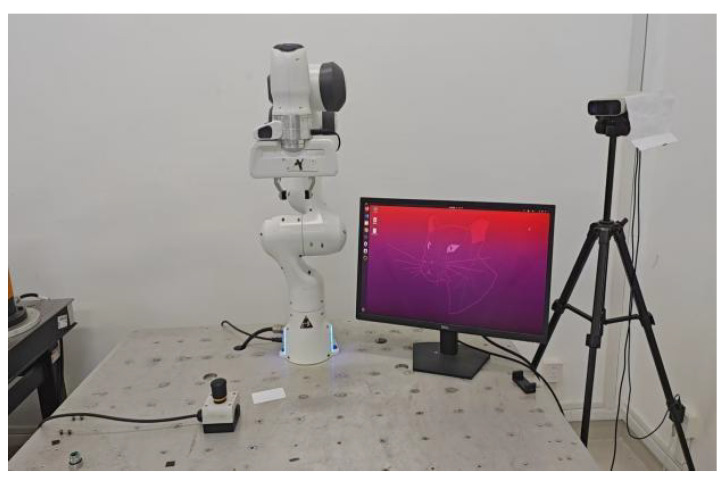
Robotic arm experimental platform.

To evaluate the performance of the proposed torque estimation algorithm, experiments were conducted on the Franka robotic arm, as illustrated in Figure 1, chosen as the platform for collision detection and external torque estimation. Developed by Franka Emika in Germany, Franka integrates high precision, flexibility, and safety, and is widely employed in research and industry. Each joint is equipped with a high-resolution torque sensor (<0.05 N), making it particularly suitable for experimental validation.

**Figure 2:**
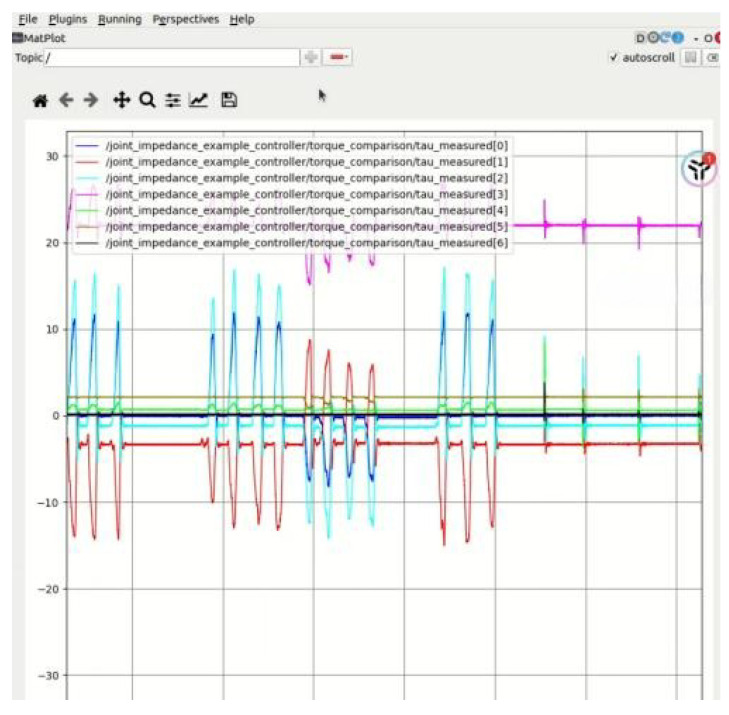
Corresponding sensor data recorded during the interaction.

The experimental protocol comprised three scenarios: torque estimation under fixed-configuration, no-load conditions to assess noise suppression; rigid-box impacts at the end-effector to evaluate collision detection; and transient human hand interactions to examine external torque estimation under compliant contact. Given that the proposed algorithm relies on the assumption of a known dynamic model, a comprehensive dynamic identification of the Franka manipulator was performed prior to the collision detection experiments to ensure the validity and reliability of the subsequent evaluations. In Figure 2, the data acquisition process is illustrated. The joint torque sensors embedded in each link of the robotic arm capture the measured torques experienced by the joints during motion.

For ease of reading, an abbreviation table for the algorithms is provided, as shown in Table 1. External torque of the Franka arm under collision-free conditions was estimated using ESO, KFMO, SH-AKFMO, and VBKFMO-Q.

The initial value of the process noise is set as follows: $Q_{0}=\left[\begin{array}{cc} Q_{cf} & 0\\ 0 & Q_{cf} \end{array}\right]$. The observation noise covariance $R_{c}=MQ_{\dot{q}}M^{T}$ is set to the current actual value. The initial values $u_{0}=n_{x}+2$ and $n_{x}$ is set to 8. Main parameters of ESO: $\beta_{01}=80$, $\beta_{02}=3200$. Main parameters of KF: $Q_{cf}$ = 80,000I. Main parameters of SH-AKF: $Q_{cf}$ = 40,000I, λ=0.99. Main parameters of AETE: $Q_{cf}$ = 80,000I, N=10, λ=0.99.

**Figure 3:**
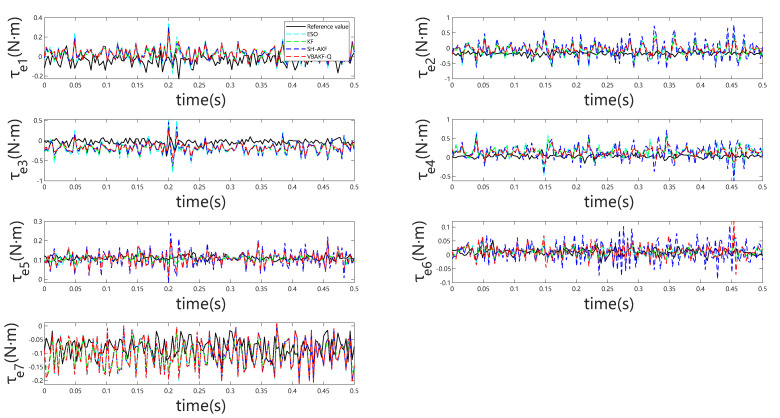
Joint torque estimates for all seven joints under zero external torque.

**Figure 4:**
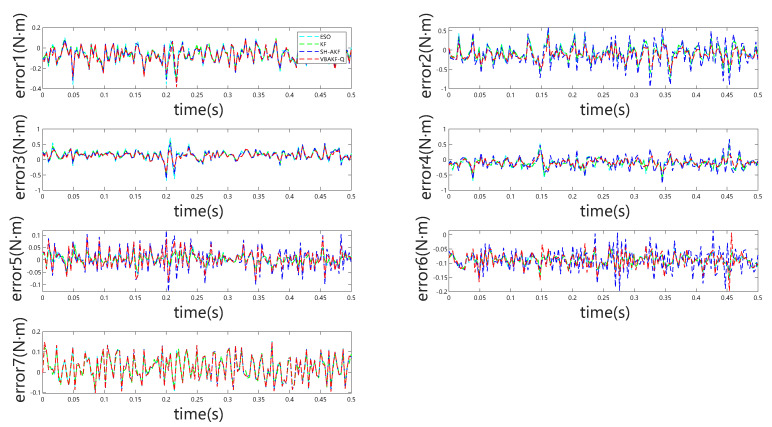
Joint torque estimation errors for all seven joints under no external torque.

Figure 3 indicates that, even in the absence of external forces, all considered momentum observers exhibit non-negligible estimation bias arising from modeling inaccuracies. During external torque estimation, abrupt transients may be erroneously interpreted as collisions. However, the observations presented in Figure 4 reveal that the absolute estimation errors remain low across the different observers, minimizing the likelihood of false collision detection. This pattern is further substantiated by the results summarized in Table 2.

**Figure 5:**
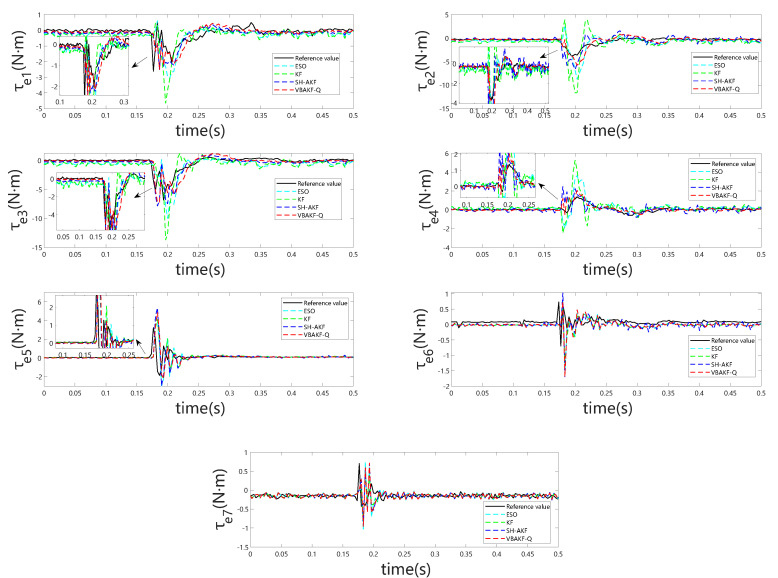
oint torque estimation for all seven joints under rigid object collision.

**Figure 6:**
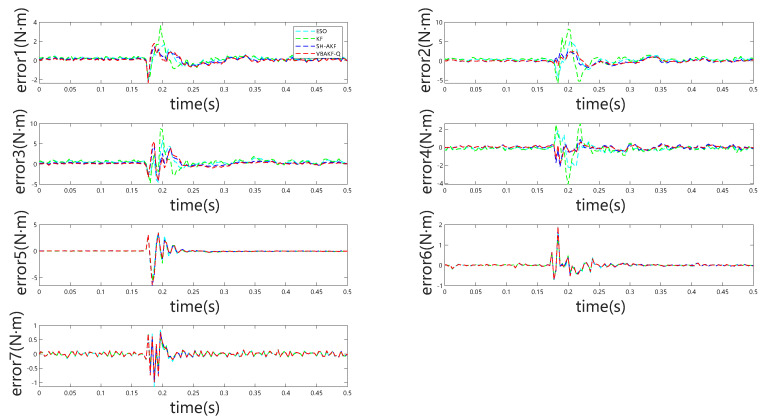
Joint torque estimation errors for all seven joints under rigid object collision.

Figure 5 and Figure 6 depict the observer outputs and corresponding joint-wise external torque estimation errors during collisions with a rigid object. Notably, for joints 1 through 4, ESO and KF exhibit substantial overshoot and discernible phase lag, whereas joints 5 through 7 show comparable performance across all four observers. This discrepancy arises because the collision occurs near the end-effector, with proximal joints (5–7) experiencing minimal perturbation, resulting in estimation behavior akin to collision-free conditions. From the detailed visualization of torque estimation, it is evident that the red dashed curve exhibits greater smoothness, and the predicted results align more closely with the actual torque.

In Table 3, evaluation based on root-mean-square error indicates that VBKFMO-Q achieves marginally superior external torque estimation relative to SH-AKFMO, while ESO and KF demonstrate considerably lower accuracy.

**Figure 7:**
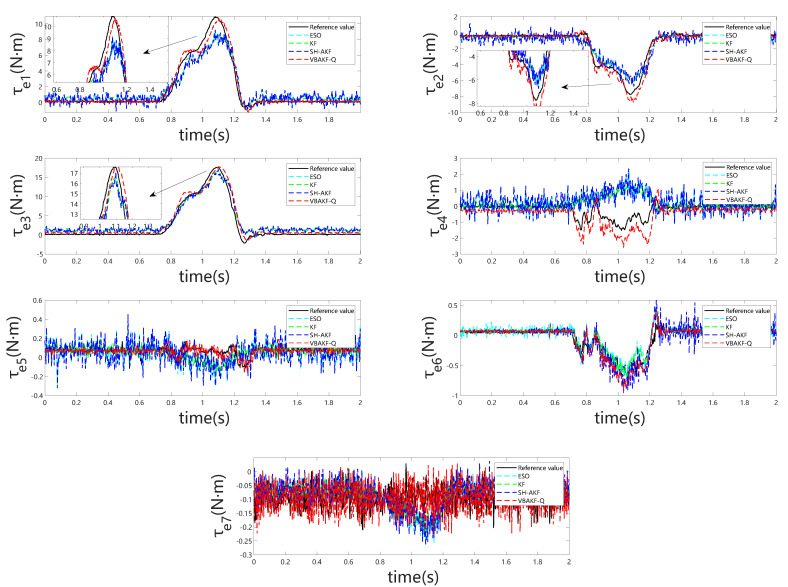
Joint torque estimates during interaction with a soft object (human hand).

**Figure 8:**
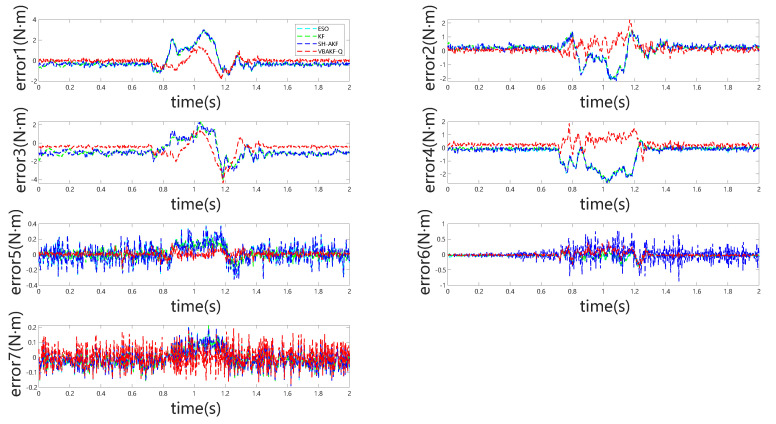
Joint torque estimation errors during interaction with a soft object (human hand).

Figure 7 depicts joint torque estimation during a brief (0.5 s) interaction with a compliant object (human hand) at the robotic arm’s end-effector, showing the external torque observations from the different observers. When the robotic manipulator collides with a compliant object, the advantages of VBKFMO-Q become particularly pronounced, especially in the first three joints, where it demonstrates markedly superior torque estimation accuracy and response speed compared to other torque observers of the same class. The corresponding joint-wise estimation error curves are presented in Figure 8, and Table 4 summarizes the root-mean-square errors for each observer throughout this interaction. Notably, the red dashed line represents the proposed VBKFMO-Q observer, which exhibits superior rapid response and estimation accuracy under soft contact conditions, closely approximating the actual external torque. Its smooth output profile further underscores its suitability for practical engineering applications.

Root-mean-square error analysis demonstrates that, in line with the preceding two experimental scenarios, the proposed algorithm consistently yields lower RMSE, with torque trajectories exhibiting both smoothness and rapid dynamic response. A consistent observation across all three experiments is that, given end-effector contact, torque estimations and corresponding RMSE progressively smooth with increasing joint indices. This phenomenon reflects that, under sufficiently accurate modeling, joints distal to the collision site are subject to attenuated perturbations, thereby providing a rigorous basis for subsequent collision isolation and identification.

## 4. Conclusions

In this work, an adaptive external torque estimation algorithm for robotic manipulators was proposed, capable of automatically adjusting the system process noise covariance matrix. First, commonly used generalized momentum observers were reviewed and derived based on the robot dynamics model to address human–robot safety requirements. Then, an external torque estimation method grounded in generalized momentum was described, and the effects of covariance mismatch on Kalman filter estimation were analyzed. To handle practical situations where the system process noise is unknown and difficult to model, a method was developed to adaptively adjust the process noise covariance matrix using historical data, which was then incorporated into the Kalman filter recursive equations to improve estimation accuracy. The algorithm was validated experimentally on the Franka manipulator under three scenarios: static with no external torque, collision with a rigid object, and brief contact with a compliant object. The proposed algorithm is broadly applicable to serial-link manipulators with identified dynamics, ensuring compatibility with widely used robotic systems. The results demonstrate that the proposed method provides fast and accurate external torque estimates without additional sensors, offering a reliable basis for safe operation in human–robot collaborative tasks.

## Figures and Tables

**Table 1:** Abbreviations.

Abbreviation	Full Name	Description (Optional)
ESO	Extended State Observer	Observer estimating system states and total disturbances
KFMO	Kalman Filter-based Momentum Observer	Observer estimating external torques using momentum dynamics and Kalman filtering
SH-AKFMO	Sage–Husa adaptive Kalman filtering-based Momentum Observer	Observer that estimates external torques by integrating momentum dynamics with Sage–Husa adaptive Kalman filtering
VBKFMO-Q	Variational Bayesian Kalman Filter-based Momentum Observer with adaptive *Q*	Observer estimating external torques using momentum dynamics and a variational Bayesian Kalman filter with adaptive process noise covariance

**Table 2:** Joint torque estimation RMSE under collision-free conditions.

Algorithm	ESO	KFMO	SH-AKFMO	VBKFMO-Q
RMSE(J1)	0.1091	0.0935	0.1068	0.1045
RMSE(J2)	0.085	0.0819	0.1314	0.1261
RMSE(J3)	0.2358	0.1827	0.2113	0.1773
RMSE(J4)	0.2844	0.1166	0.0975	0.1001
RMSE(J5)	0.032	0.0166	0.0478	0.0377
RMSE(J6)	0.1105	0.0895	0.0894	0.0896
RMSE(J7)	0.0692	0.0594	0.0687	0.0688

**Table 3:** Joint torque estimation RMSE during high-stiffness impacts.

Algorithm	ESO	KFMO	SH-AKFMO	VBKFMO-Q
RMSE(J1)	1.3122	1.3756	0.4347	0.331
RMSE(J2)	2.9709	2.7695	1.4114	1.3468
RMSE(J3)	2.4096	2.1156	1.0478	1.1456
RMSE(J4)	0.8097	0.8055	0.2118	0.1577
RMSE(J5)	0.429	0.4187	0.4582	0.4479
RMSE(J6)	0.1144	0.099	0.1173	0.1154
RMSE(J7)	0.1181	0.0917	0.1124	0.1143

**Table 4:** Joint torque estimation RMSE during compliant contact (human hand).

Algorithm	ESO	KFMO	SH-AKFMO	VBKFMO-Q
RMSE(J1)	1.0266	0.8956	1.0259	0.286
RMSE(J2)	0.8367	0.6369	0.8364	0.4485
RMSE(J3)	1.3626	1.2528	1.3612	1.0145
RMSE(J4)	1.0637	0.9181	1.0622	0.4996
RMSE(J5)	0.1237	0.0791	0.1244	0.042
RMSE(J6)	0.1017	0.0876	0.1439	0.0968
RMSE(J7)	0.0715	0.0661	0.0737	0.0762

## Data Availability

Data available upon request from the authors. The data that support the findings of this study are available from the corresponding author, upon reasonable request.
